# Immunization with a murine cytomegalovirus based vector encoding retrovirus envelope confers strong protection from Friend retrovirus challenge infection

**DOI:** 10.1371/journal.ppat.1008043

**Published:** 2019-09-30

**Authors:** Nadine Bongard, Vu Thuy Khanh Le-Trilling, Anna Malyshkina, Meike Rückborn, Kerstin Wohlgemuth, Ina Wensing, Sonja Windmann, Ulf Dittmer, Mirko Trilling, Wibke Bayer

**Affiliations:** Institute for Virology, University Hospital Essen, University Duisburg-Essen, Essen, Germany; Vaccine Research Center, UNITED STATES

## Abstract

Immunization vectors based on cytomegalovirus (CMV) have attracted a lot of interest in recent years because of their high efficacy in the simian immunodeficiency virus (SIV) macaque model, which has been attributed to their ability to induce strong, unusually broad, and unconventionally restricted CD8^+^ T cell responses. To evaluate the ability of CMV-based vectors to mediate protection by other immune mechanisms, we evaluated a mouse CMV (MCMV)-based vector encoding Friend virus (FV) envelope (Env), which lacks any known CD8^+^ T cell epitopes, for its protective efficacy in the FV mouse model. When we immunized highly FV-susceptible mice with the Env-encoding MCMV vector (MCMV.env), we could detect high frequencies of Env-specific CD4^+^ T cells after a single immunization. While the control of an early FV challenge infection was highly variable, an FV infection applied later after immunization was tightly controlled by almost all immunized mice. Protection of mice correlated with their ability to mount a robust anamnestic neutralizing antibody response upon FV infection, but Env-specific CD4^+^ T cells also produced appreciable levels of interferon γ. Depletion and transfer experiments underlined the important role of antibodies for control of FV infection but also showed that while no Env-specific CD8^+^ T cells were induced by the MCMV.env vaccine, the presence of CD8^+^ T cells at the time of FV challenge was required. The immunity induced by MCMV.env immunization was long-lasting, but was restricted to MCMV naïve animals. Taken together, our results demonstrate a novel mode of action of a CMV-based vaccine for anti-retrovirus immunization that confers strong protection from retrovirus challenge, which is conferred by CD4^+^ T cells and antibodies.

## Author summary

CMV-based vectors have attracted a lot of attention in the vaccine development field, since they were shown to induce unconventionally restricted CD8^+^ T cell responses and strong protection in the SIV rhesus macaque model. In a mouse retrovirus model, we show now that immunization with a mouse CMV-based vector encoding retrovirus envelope conferred very strong protection, even though it was not designed to induce any CD8^+^ T cell responses. In this MCMV.env immunization, protection relied on the induction of CD4^+^ T cells and the ability to mount a strong anamnestic neutralizing antibody response upon retrovirus infection, but it was restricted to MCMV pre-naïve mice. In our model system, the MCMV based vector shows very high efficacy that is comparable to an attenuated retrovirus-based vaccine, and encourages the pursuit of this vaccination strategy.

## Introduction

In the last two decades, vector-based immunization approaches for the development of an HIV vaccine have been pursued intensively, and recently vectors based on cytomegalovirus (CMV) have drawn a lot of interest. At first glance, CMV is not an obvious choice as basis for a vaccine vector: as a β-herpes virus it carries a large and highly complex genome [1] that encodes numerous immune evasion proteins interfering with many aspects of immunity [2], and CMV infection is associated with severe illness in immune compromised or immature patients [3]. However, after a long period of productive replication following the primary infection, CMV establishes latency from which repetitive episodes of virus reactivation can occur, leading to recurrent rounds of immunogen expression and creating a self-boosting vaccine. Furthermore, the natural CMV infection can induce inflationary T cell responses, which do not contract after the effector phase but keep expanding and can reach very high frequencies (reviewed in [4, 5]), maybe a desirable feature of vaccine-induced immunity.

In recent years, CMV-based vectors for immunization have drawn increasing interest. There have been a number of approaches evaluating the murine CMV (MCMV) as a vaccine vector in mice. For the induction of CD8^+^ T cell based immunity, epitope-based vaccines have been constructed using epitopes from influenza virus [6], lymphocytic choriomeningitis virus [6] or Ebola virus [7] as sole immunogens, which induced strong immune responses and protection in the respective challenge models. For immunization against Mycobacterium tuberculosis, an MCMV vector encoding a tetanus toxin fragment was tested in a mouse model and was found to induce an antibody-dominated response [8]. Similarly, a rhesus CMV (RhCMV) based vaccine encoding an Ebola virus glycoprotein conferred protection to macaques from Ebola virus challenge but induced mainly antibody and not cellular immune responses [9]. Finally, RhCMV-based vectors were developed in the simian immunodeficiency virus (SIV) infection model in non-human primates and were shown to confer very strong protection in half of the vaccinated monkeys [10]. Interestingly, RhCMV-based immunization induced very broad CD8^+^ T cell responses to epitopes presented on major histocompatibility complex (MHC) type II and MHC-I E [11, 12], which was caused by deletion of multiple genes in this RhCMV vector [11, 13].

To evaluate the potential of CMV-based immunization when neither vector design nor immunogen choice are targeted at the induction of CD8^+^ T cell responses, we constructed a vaccine vector based on MCMV that we employed in a mouse retrovirus model. Friend retrovirus (FV) is a murine retrovirus complex consisting of the apathogenic, replication-competent Friend murine leukemia virus (F-MuLV) and the replication-defective but pathogenic spleen focus forming virus (SFFV; [14]). Infection of susceptible mice results in the rapid development of splenomegaly and erythroleukemia due to an aberrant activation of the erythropoietin receptor by the SFFV envelope protein gp55, whereas mice that are genetically resistant to FV-induced disease develop a chronic infection (reviewed in [15]).

FV is regarded as a useful retrovirus mouse model that allows for insights into immunological control of retrovirus infections in general, and it shows similarities to HIV infection with regard to the establishment of persistent reservoirs in immunologically privileged sites [16] as well as immunosuppression driven by regulatory T cells [17, 18]. As low doses of FV are sufficient to rapidly induce disease in susceptible mice, FV infection is a very stringent mouse model for the development and evaluation of immunization strategies. We and others have employed the FV model extensively in the past to develop improved immunization strategies and to analyse mechanisms underlying protection conferred by different vaccines against retroviruses. Vaccines based on attenuated F-MuLV or FV [19–23], inactivated F-MuLV [24], protein or peptide vaccines [25–28], nanoparticle-based vaccines [29] and vector-based vaccines [30–40] have been tested and shown to confer widely different degrees of protection. The most potent vaccine in the FV model described until now is live-attenuated F-MuLV, which completely protects even highly susceptible mice from FV infection [21]. It has been demonstrated that a complex immune response comprising antibodies as well as CD4^+^ and CD8^+^ T cells is necessary for this protection [19, 20]. Interestingly, we could demonstrate in adenovirus-based vaccine studies that very potent, albeit not sterile, protection can also be conferred if only individual immune components are induced, as the induction of strong CD8^+^ T cell responses will also protect highly susceptible mice from FV-induced disease and allow them to control FV infection at a very low level [35]. In a side-by-side comparison we showed that the refined employment of adenovirus-based vaccines mediated protection from FV infection that was almost as strong as that conferred by immunization with attenuated F-MuLV. However, the mechanisms underlying protection conferred by the two vaccines differed significantly, as the adenovirus-based vector induced T cell dominated responses, whereas the F-MuLV immunization induced little T cell responses but highly superior antibody responses [40].

To evaluate the general potency of MCMV-based immunization in the FV model, we constructed an MCMV vector encoding F-MuLV envelope, without introducing any modifications into the vector aiming at unconventionally restricted CD8^+^ T cell induction. In the work presented here, we show that this envelope-encoding MCMV-based vaccine confers strong protection in the FV mouse model, without any evidence for a contribution of vaccine-induced CD8^+^ T cells.

## Results

### Characterization of the MCMV.env vector

To analyse the potential of CMV-based vectors in the FV mouse model, we constructed a vector based on mouse cytomegalovirus (MCMV) encoding the envelope protein (Env) of Friend murine leukemia virus (F-MuLV) and tested its efficacy at preventing FV infection of highly susceptible CB6F1 mice. To prevent rapid control of the MCMV vector by natural killer cells, the *m157* coding sequence was partially deleted, leaving the neighbouring open reading frames intact [41], and replaced by the Env transgene expression cassette. An MCMV vector on the *m157*-deleted background without any transgene was used as a control vector.

**Fig 1:**
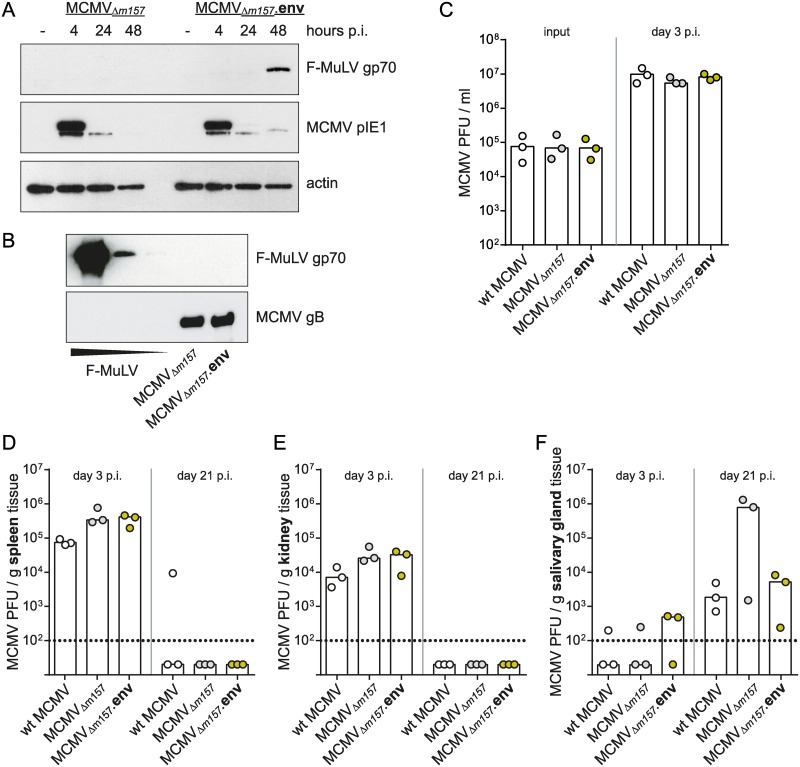
Characterization of MCMV.env. (A) Expression of F-MuLV Env from the MCMV.env vector was verified by immunoblot analysis of lysates of MEF cells infected with the MCMV_Δ*m157*_-based vector MCMV.env or the non-transgene encoding control vector collected 4, 24 or 48 hours after infection. Uninfected MEF cells served as negative control, detection was performed with antibodies directed against the indicated proteins. (B) 2 × 10^5^ PFU MCMV.env or MCMV, or different amounts of F-MuLV particles (1 × 10^5^ FFU, 5 × 10^3^ FFU, or 1 × 10^3^ FFU) were subjected to SDS-PAGE and Western Blot. Presence of F-MuLV Env gp70 was probed with gp70-specific polyclonal antibody, detection of MCMV gB served as loading control. (C-F) For characterization of the growth kinetics of the MCMV_Δ*m157*_-based vector MCMV.env in comparison to non-transgene encoding vector or to wildtype MCMV, *in vitro* (C) or *in vivo* (D-F) replication studies were performed. (C) MEF cells were infected with the indicated viruses, and plaque forming units (PFU) of input virus as well as of supernatants collected three days p.i. were analysed. (D-F) CB6F1 mice were infected with 2 × 10^5^ PFU of the indicated viruses, and MCMV titers in spleen (D), kidney (E) and salivary gland tissue (F) were analyzed three and 21 days after infection. Data were obtained in one experiment, each dot indicates one infection (C) or one mouse (D-F), bars indicate median values. Data were analysed for statistically significant differences by One Way ANOVA on Ranks with the Dunn’s post test; no significant differences were observed.

The expression of F-MuLV Env in MCMV.env infected cells was verified by immunoblot analysis (Fig 1A). The presentation of immunogens on the vaccine vector particles can be beneficial for the induction of immunogen-specific antibody responses [32], therefore we also analysed if Env is incorporated into the MCMV.env virions. However, the analysis of purified MCMV.env particles by immunoblot gave no indication of incorporation of significant amounts of Env protein (Fig 1B).

A comparison of *in vitro* replication showed comparable progeny titers for wildtype (wt) MCMV, the *m157*-deleted control vector and MCMV.env (Fig 1C). To compare the *in vivo* replication rates, we infected CB6F1 mice with 2 x 10^5^ PFU of the *m157*-deleted control vector or MCMV.env, or wt MCMV, and analysed MCMV titers in spleen, kidney and salivary gland tissue on day 3 and day 21 after infection. While the titers of the *m157*-deleted vectors were slightly higher on day 3 in spleen and kidney tissues compared to wt MCMV, they were mostly cleared from these tissues on day 21 p.i. (Fig 1D and 1E). The titers in salivary glands on day 21 p.i. showed higher variability but seemed comparable for wt MCMV and the *m157*-deleted MCMV.env, whereas 2 of 3 samples showed higher titers for the *m157*-deleted control vector (Fig 1F). Taken together, these data suggest that the deletion of *m157* does not severely influence pathogenicity of MCMV in CB6F1 mice that we used in the subsequent vaccination experiments.

**Fig 2:**
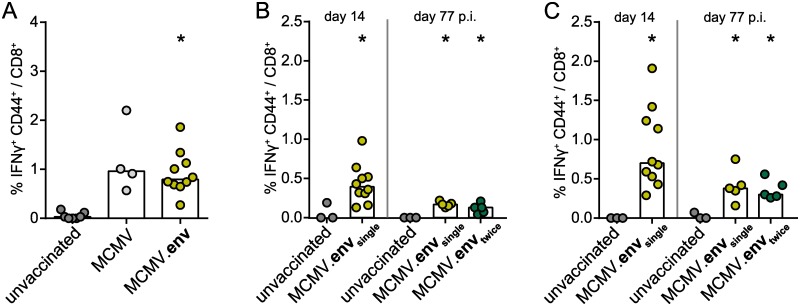
MCMV-specific CD8^+^ T cell response. CB6F1 mice were infected once or twice in a three-week interval with 2 × 10^5^ PFU of the indicated MCMV-based vectors. (A) Two weeks after a single immunization, the MCMV-specific CD8^+^ T cell response was analysed after restimulation with a pool of five MCMV-derived peptides. (B, C) Two weeks or 11 weeks after the first immunization, the MCMV-specific CD8^+^ T cell response was analysed after restimulation with a pool of four peptides representing the conventional MCMV-epitopes M45_985-993_, M57_816-824_, M102_446-455_ and m141_15-23_ (B) or with a peptide representing the inflationary MCMV epitope m139_419-426_ (C). Data were acquired in two (A) or one experiments (B, C), each dot represents one mouse, bars indicate mean values. Statistically significant differences compared to unvaccinated mice are indicated by * (*P* < 0.05; One Way ANOVA, Holm-Sidak post test).

When we analysed the MCMV-specific immune response, we found that mice infected with either of the *m157*-deleted vectors mounted comparable MCMV-specific CD8^+^ T cell responses (Fig 2A). An analysis of CD8^+^ T cells specific for conventional (M45_985-993_, M57_816-824_, M102_446-455_ and m141_15-23_; Fig 2B) or inflationary epitopes (m139_419-426_; Fig 2C) 14 days or 77 days after a single or repeated infection with MCMV.env revealed a sustained MCMV-specific CD8^+^ T cell response, with no significant difference between the single or the repeat administration. Interestingly, the MCMV-specific CD8^+^ T cell response was fairly low overall, which may be characteristic for this particular mouse strain.

### A single immunization with MCMV.env confers strong protection from delayed FV challenge infection

**Fig 3:**
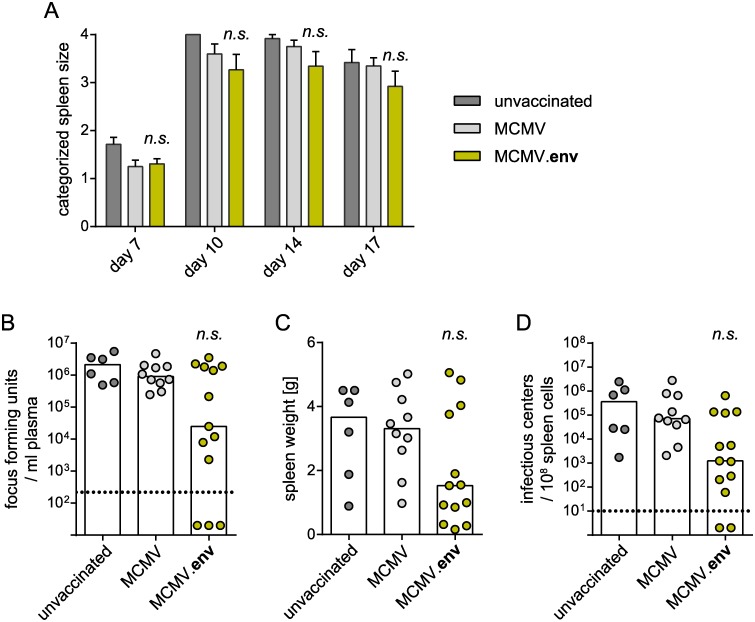
No protection from early FV challenge after MCMV.env immunization. CB6F1 mice were immunized once with MCMV.env, or a non-transgene-encoding control MCMV, and infected with 5 000 SFFU FV three weeks later. Development of splenomegaly was monitored by twice-weekly palpation of the spleens (A). Viremia levels were determined in blood samples collected 10 days after FV infection (B); spleens were isolated three weeks after FV infection and spleen weights (C) and spleen viral loads were analysed (D). Each dot indicates an individual mouse, columns indicate mean (A, C) or median values (B, D), column whiskers indicate the standard error of the means (A), dotted lines indicate the limits of detection. Data were obtained in three independent experiments, using 6 (unvaccinated), 10 (MCMV) or 13 mice (MCMV.env) per group. Data were analysed for statistically significant differences by One Way ANOVA on Ranks with the Dunn’s post test (n.s.: not significant).

To analyse the protective potential of the new MCMV.env construct, we immunized CB6F1 mice with 2 x 10^5^ PFU MCMV.env, or the MCMV vector without transgene as a control, and infected them with FV three weeks later to analyse the protection from FV challenge. However, the immunized mice did not show an improved control over FV induced splenomegaly compared to unvaccinated mice (Fig 3A), and we did not observe any significant differences in overall viremia levels on day 10 after FV infection (Fig 3B) or in spleen weights (Fig 3C) or spleen viral loads on day 21 after FV challenge infection (Fig 3D), even though individual mice were able to control the FV infection.

**Fig 4:**
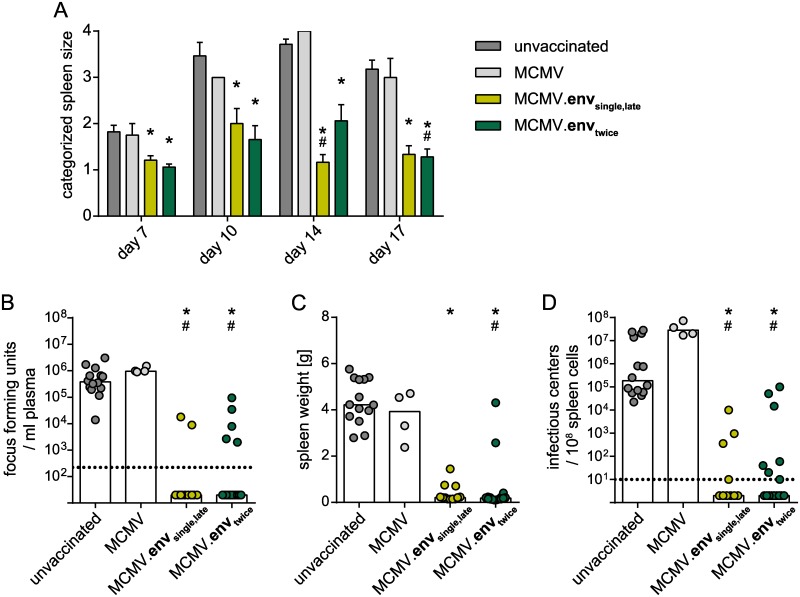
Strong protection from late FV challenge after single or repeat MCMV.env immunization. CB6F1 mice were immunized with MCMV.env, or a non-transgene-encoding control MCMV, either once, or twice in a three-week time interval, and infected with 5 000 SFFU FV six weeks after the first immunization. Development of splenomegaly was monitored by twice-weekly palpation of the spleens (A). Viremia levels were determined in blood samples collected 10 days after FV infection (B); spleens were isolated three weeks after FV infection and spleen weights (C) and spleen viral loads were analysed (D). Each dot indicates an individual mouse, columns indicate mean (A, C) or median values (B, D), column whiskers indicate the standard error of the means (A), dotted lines indicate the limits of detection. Data were obtained in two (MCMV), three (MCMV.env_single,late_) or five independent experiments (MCMV.env_twice_, unvaccinated), using 4 (MCMV), 12 (MCMV.env_single,late_) or 14 mice (unvaccinated, MCMV.env_twice_) per group. Statistically significant differences compared to unvaccinated mice are indicated by *, significant differences compared to mice vaccinated with the MCMV control vector are indicated by # (*P* < 0.05, One Way ANOVA on Ranks, Dunn’s post test).

Seeking to improve the vaccination efficacy, we evaluated the application of a second immunization, and repeated the MCMV.env immunization three weeks after the first immunization (MCMV.env_twice_). Since productive MCMV replication is observed for a long period after primary infection (> 21 days; Fig 1F) that should lead to long-lasting immunogen expression, we also included a group of mice that was immunized only once, but challenged with FV at the delayed time point of 6 weeks post immunization (MCMV.env_single,late_). Surprisingly, when mice were infected with FV six weeks after the start of the immunization regimen, both groups of MCMV.env immunized mice controlled the infection equally well. All MCMV.env immunized mice had significantly smaller spleens than unvaccinated mice or mice vaccinated with the control MCMV throughout the observation period (Fig 4A). While unvaccinated mice or the control MCMV immunized mice had high viremia levels at day 10 after FV challenge infection, most MCMV.env immunized mice of either group had no detectable viremia (Fig 4B). Similarly, when the spleens were collected three weeks after FV infection, the MCMV.env immunized mice had significantly smaller spleens than unvaccinated mice, with the spleens of most of these mice exhibiting a normal weight (Fig 4C). The viral loads in spleens of unvaccinated mice and of mice immunized with the control MCMV were equally high, whereas the spleen viral loads of mice from either MCMV.env immunized group were significantly reduced in comparison to both unvaccinated and control MCMV immunized mice, and FV was actually undetectable in many mice (Fig 4D).

These results demonstrate that a single immunization with MCMV.env confers strong protection against a delayed FV challenge infection, and that in this delayed challenge setting, the single immunization regimen is not inferior to a repeat immunization schedule.

### Improved anamnestic antibody responses after late FV challenge infection

**Fig 5:**
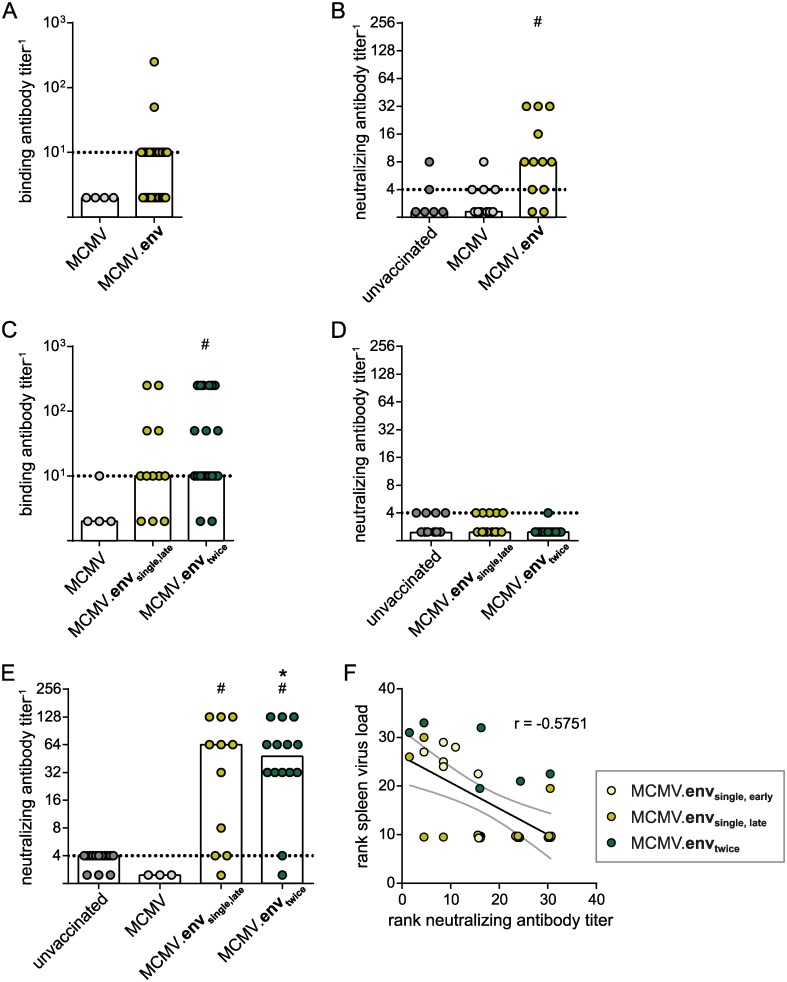
Antibody response to MCMV.env immunization. CB6F1 mice were immunized with MCMV.env, or a non-transgene-encoding control MCMV, either once, or twice in a three-week time interval. Binding antibody responses were analysed two weeks after the first immunization (A) and five weeks after the first immunization (C). Neutralizing antibody responses were analysed ten days after FV challenge infection in the early challenge setting (B), five weeks after the first immunization in the late-challenge setting (D) or ten days after infection in the late-challenge setting (E). (F) Spearman ranked correlation analysis showing ranked spleen viral loads shown in Figs 3D and 4D and ranked neutralizing antibody titers shown in (B) and (E). Dots representing identical rank values were off-set for visualization, but analysis of correlation was performed with original, identical values. Each dot indicates an individual mouse, columns indicate median values, dotted lines indicate the limits of detection, grey lines indicate the 95% confidence interval. Data were obtained in two (A, C, D: MCMV), three (B; A, C, D: MCMV.env_single,late_) or five independent experiments (A, C, D: MCMV.env_twice_; unvaccinated). Statistically significant differences compared to unvaccinated mice are indicated by *, significant differences compared to mice vaccinated with the MCMV control vector are indicated by # (*P* < 0.05, One Way ANOVA on Ranks, Dunn’s post test).

Seeking for an explanation for the improved protection after delayed FV challenge infection, we analysed the immune responses to the different vaccination regimens. Analysing the antibody responses to MCMV.env immunization, we found that two weeks after a single immunization, binding antibody levels were rather low and at the limit of detection in about half of the mice; only two mice showed higher antibody levels (Fig 5A). When mice were challenged early after the single immunization, the anamnestic neutralizing antibody response ten days after FV challenge was low, albeit significantly improved compared to unvaccinated mice (Fig 5B). In the prolonged vaccination regimen, mice that were immunized once and mice that had received a repeat immunization had comparable binding antibody levels five weeks after initiation of the immunization, but they were still rather low with median values at the detection limit (Fig 5C). Only very low neutralizing antibody responses were detected in few immunized mice at this time point (Fig 5D). However, most mice of both immunized groups were able to mount a robust neutralizing antibody response ten days after FV challenge infection that was significantly higher than in unvaccinated mice or in mice that were immunized with the control MCMV (Fig 5E).

These findings suggest that the ability of mice to mount a neutralizing antibody response after FV infection may be crucial for the MCMV.env mediated protection, and that the short time period to the early FV challenge is insufficient for the maturation of the antibody response. In fact, a Spearman ranked correlation analysis of the data obtained for the FV challenge 3 weeks or 6 weeks after MCMV.env immunization suggests an inverse correlation of neutralizing antibody levels 10 days after FV challenge with spleen viral loads 21 days after FV challenge (r = -0.5751; *P* = 0.0005; Fig 5F).

### Immunization with MCMV.env induces strong Env_123-141_-specific CD4^+^ T cell responses

**Fig 6:**
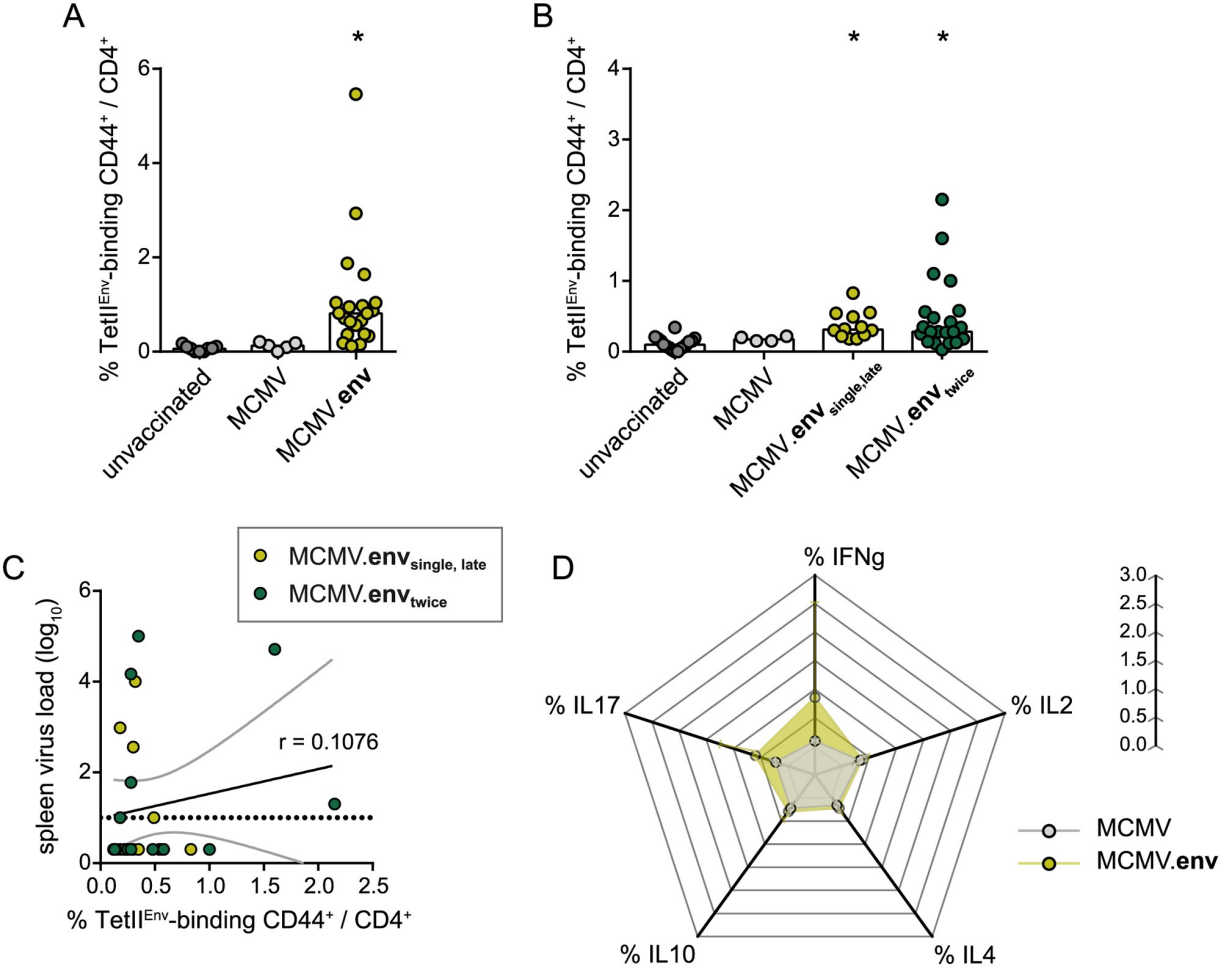
MCMV.env immunization leads to induction of strong Env_123-141_-specific CD4^+^ T cell responses. CB6F1 mice were immunized with MCMV.env, or a non-transgene-encoding control MCMV, either once, or twice in a three-week time interval, and Env-specific immune responses were analysed in peripheral blood cells by MHC II tetramer staining two weeks after the first immunization (A) or five weeks after the first immunization (B). (C) Correlation analysis of Env-specific CD4^+^ T cells shown in (B) and viral loads in spleens of immunized mice shown in Fig 4D. (D) Two weeks after the first immunization, cytokine production of CD4^+^ T cells from peripheral blood was analysed after *in vitro* restimulation with a pool of Env-derived peptides. (A-C) Each dot indicates an individual mouse, columns indicate the mean values, dotted line indicates the detection limit, grey lines indicate the 95% confidence interval. (A) Data were obtained in two (MCMV) or five independent experiments (MCMV.env, unvaccinated), using 5 (MCMV), 9 (unvaccinated) or 21 mice (MCMV.env) per group. (B) Data were obtained in two (MCMV), three (MCMV.env_single,late_) or five independent experiments (MCMV.env_twice_, unvaccinated), using 4 (MCMV), 12 (MCMV.env_single,late_) or 14 mice (unvaccinated, MCMV.env_twice_) per group. (D) Data were obtained in one experiment; dots indicate mean values, whiskers indicate the standard deviation. Statistically significant differences compared to unvaccinated mice are indicated by * (*P* < 0.05, One Way ANOVA on Ranks, Dunn’s post test).

To determine the cellular immune responses underlying the MCMV.env mediated protection, we analysed the induction of Env_123-141_-specific CD4^+^ T cells by MHC II tetramer staining. Already two weeks after the first immunization, MCMV.env immunized mice had mounted a clearly detectable Env_123-141_-specific CD4^+^ T cell response (Fig 6A), that was absent in the control MCMV immunized mice. When CD4^+^ T cell responses were analysed three weeks later, we could still detect significant levels of Env_123-141_-specific CD4^+^ T cells after the single immunization (Fig 6B), and there was no significant difference in the Env_123-141_-specific CD4^+^ T cell response in mice that had received a second immunization three weeks after the first immunization.

Interestingly, when we performed a Spearman ranked correlation analysis, we found only a poor correlation between the frequency of Env-specific CD4^+^ T cells determined one week before FV challenge infection and the viral load in spleens 3 weeks after FV challenge infection (r = 0.1076; *P* = 0.5931; Fig 6C).

We also analysed the cytokine production of Env-specific CD4^+^ T cells after *in vitro* restimulation (Fig 6D); while responses were not very strong, we observed a trend to a higher frequency of IFNγ and IL17 producing CD4^+^ T cells in MCMV.env immunized mice compared to control MCMV immunized mice, suggesting that apart from providing help for the strong anamnestic antibody response, Env-specific CD4^+^ T cells may also exhibit some direct antiviral effector functions and provide help for CD8^+^ T cell induction.

**Fig 7:**
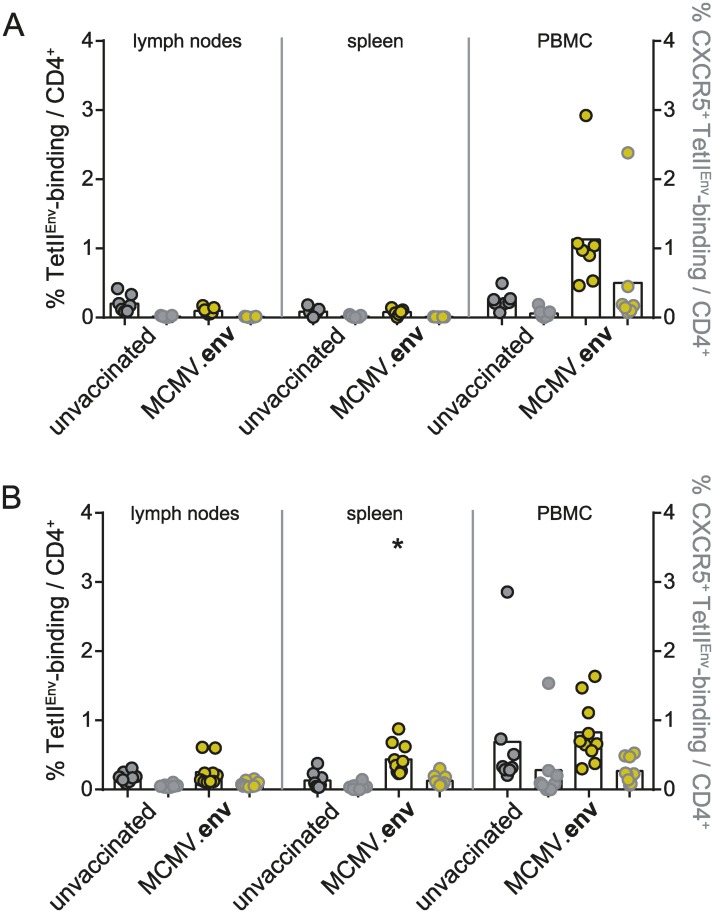
Distribution of F-MuLV-specific CD4^+^ T cells after FV challenge infection. CB6F1 mice were immunized once with MCMV.env and infected six weeks later with 5 000 SFFU FV. Two weeks after immunization (A), or three weeks after the FV infection (B), spleen, lymph node and peripheral blood mononuclear cells were collected and subjected to MHC II tetramer staining for detection of Env_123-141_-specific CD4^+^ T cells in combination with staining for the chemokine receptor CXCR5 (grey symbols). Data were obtained in two independent experiments, each dot indicates one mouse, bars indicate mean values. Data were analysed for statistically significant differences by One Way ANOVA on Ranks with Dunn’s post test. * indicates statistically significant differences compared to the respective data from unvaccinated mice (*P* < 0.05).

To analyse the localization of Env-specific CD4^+^ T cells, we collected lymph nodes, spleens and peripheral blood mononuclear cells (PBMC) from MCMV.env immunized or unvaccinated mice 14 days after MCMV.env immunization or 21 days after FV challenge infection and subjected the cells to MHC II tetramer staining to detect Env_123-141_-specific CD4^+^ T cells. After MCMV.env immunization, we detected an appreciable frequency of Env_123-141_-specific CD4^+^ T cells in PBMC of most MCMV.env immunized mice, as described above, but very low frequencies in lymph nodes and spleens (Fig 7A). We also stained the cells for expression of the chemokine receptor CXCR5 as a surrogate marker for a follicular helper phenotype and found that on average half of the Env-specific CD4^+^ T cells expressed CXCR5. After FV infection, the MHC II tetramer staining revealed similar frequencies of Env-specific CD4^+^ T cells in unvaccinated and MCMV.env immunized mice in lymph nodes and PBMC, but a significantly higher frequency in spleens (Fig 7B). Again, about half of the Env-specific CD4^+^ T cells expressed CXCR5 in MCMV.env immunized as well as in unvaccinated mice. The frequency of Env-specific CD4^+^ T cells after FV infection was lowest in lymph nodes, which carry only relatively low viral loads in FV infection [16].

### MCMV.env immunization does not induce Env-specific CD8^+^ T cells

In immunization studies using RhCMV for vaccination of rhesus macaques against SIV, unconventionally restricted CD8^+^ T cell responses were demonstrated: the RhCMV-induced CD8^+^ T cells recognized a very high number of epitopes derived from the vaccine immunogens, and they were unusual in recognising peptides presented by MHC type II or E [11, 12]. We had not introduced any modifications into the MCMV vector that have been described to be required for the induction of unconventionally restricted CD8^+^ T cell responses by RhCMV vectors [11, 13], still it was of great interest to analyse the CD8^+^ T cell response after MCMV.env immunization in more detail. In the natural FV infection, a role of CD8^+^ T cells recognising F-MuLV Env is questionable: while Ruan et al. have described an H2-D^b^ restricted CD8^+^ T cell epitope in F-MuLV Env [42] it has never been confirmed by later work, and the CD8^+^ T cell response in FV infection has been shown to be dominated by cells recognizing the Leader-Gag derived epitope GagL_85-93_ [43], which is not part of our MCMV.env vaccine.

**Fig 8:**
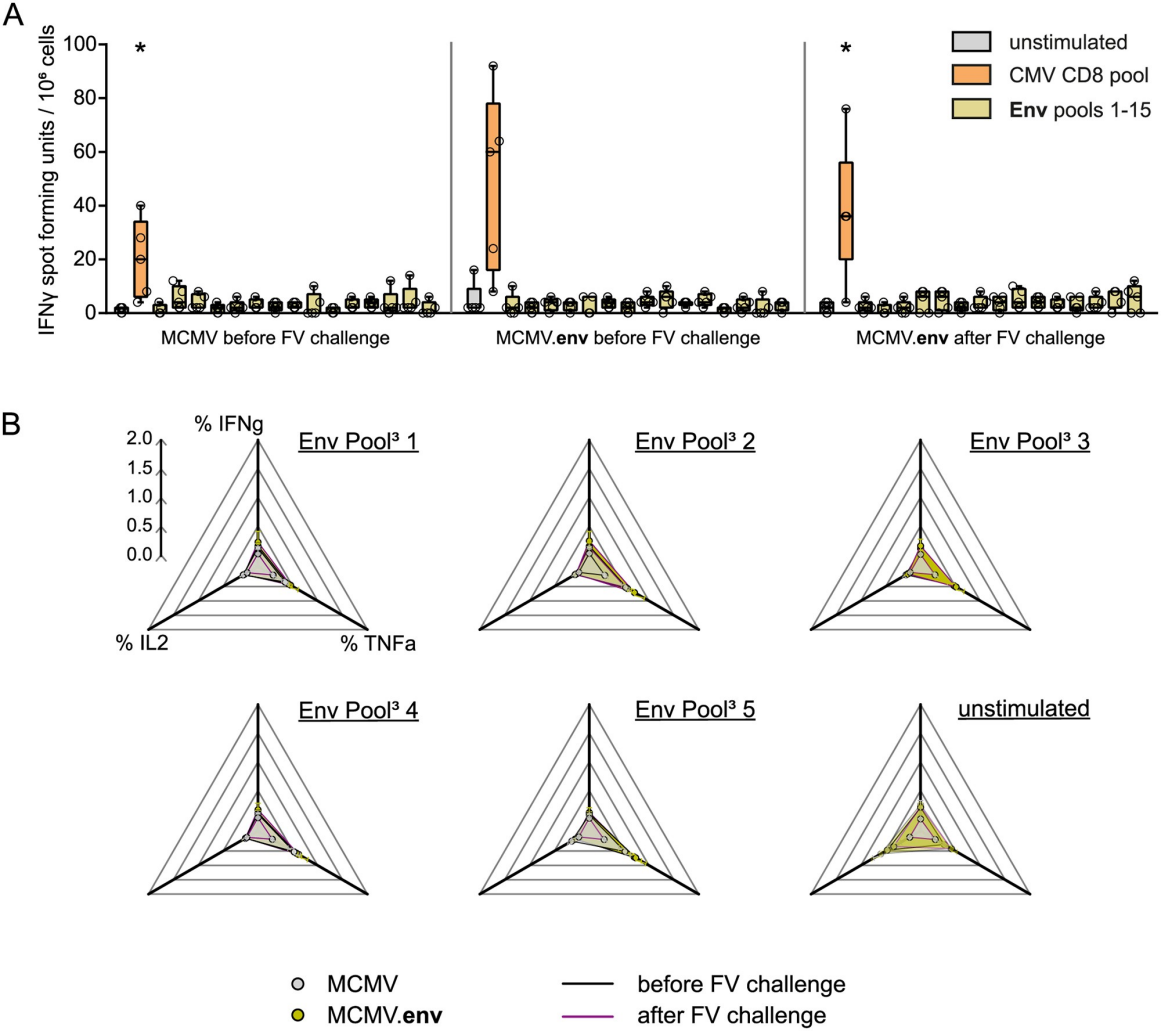
Analysis of Env-reactive T cells. CB6F1 mice were immunized once with MCMV.env, or with a non transgene-encoding MCMV as control, and infected with 5 000 SFFU FV six weeks later. Spleens were collected from mice either five weeks after immunization, or two weeks after FV infection, and restimulated *in vitro* with Env-derived peptide pools. (A) Spleen cells were depleted of CD4^+^ cells by magnetic cells sorting, and subjected to restimulation with 15 pools of overlapping peptides spanning the whole Env protein in an ELISpot assay; stimulation with a pool of CMV-derived peptides, or incubation without any peptide stimulation, served as controls. (B) Whole spleen cells were restimulated *in vitro* with pools of Env-derived peptides, and production of the indicated cytokines was analysed by flow cytometry after intracellular cytokine staining. Data were acquired in one experiment, data shown in (A) are means of two replicates. Each dot indicates an individual mouse (A) or mean values (B). Data were analysed for statistically significant differences by One Way ANOVA on Ranks with Dunn’s post test. * indicates statistically significant differences compared to unstimulated control (*P* < 0.05).

To analyse if the MCMV.env vaccine induces Env-specific CD8^+^ T cells, we performed an *in vitro* stimulation assay. We isolated spleen cells from MCMV.env or control MCMV immunized mice six weeks after immunization, or from MCMV.env immunized, FV infected mice 21 days after FV challenge, depleted the spleen cells of CD4^+^ cells, and stimulated them with Env-derived peptide pools in an IFNγ ELISpot assay, or with a pool of CMV-derived peptides as control. While the MCMV-specific CD8^+^ T cell response was readily detectable, stimulation of spleen cells with Env-derived peptide pools did not result in a significant number of IFNγ spots (Fig 8A). Of note, no response was detected to pool 8 that contains the previously described putative CD8^+^ T cell epitope [42]. These results indicate that, as intended, immunization with MCMV.env did not induce any appreciable Env-specific CD8^+^ T cell response.

Similar results were obtained when whole spleen cells from MCMV or MCMV.env immunized mice were restimulated with Env-derived peptide pools and production of various cytokines was analysed by flow cytometry. There was no appreciable production of IFNγ, TNFα or interleukin 2 (IL2) by CD8^+^ T cells of MCMV.env immunized mice either before or after FV infection (Fig 8B).

**Fig 9:**
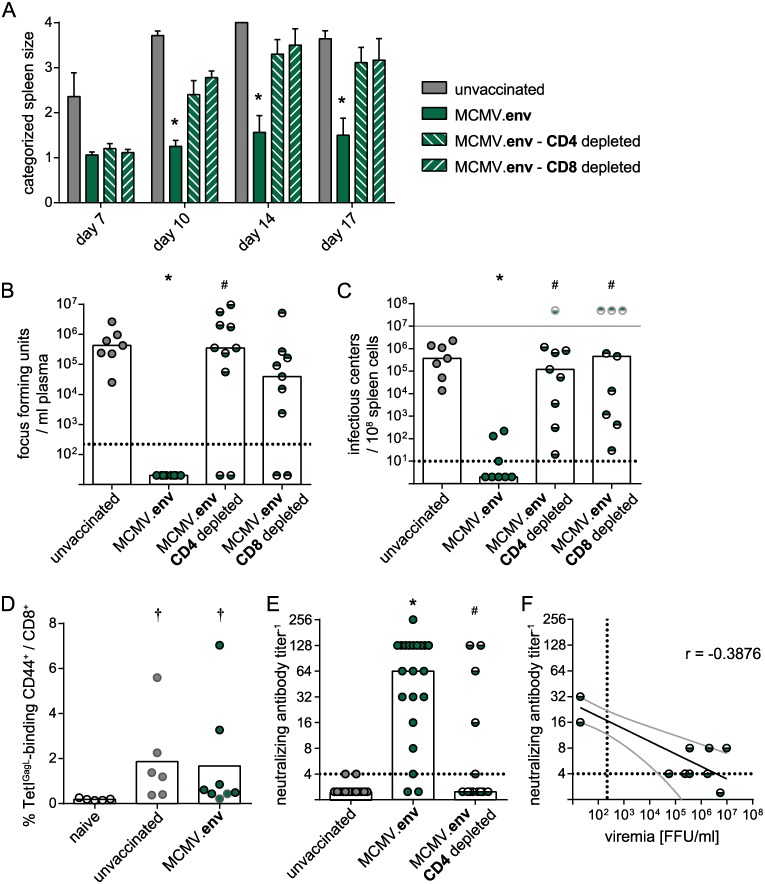
Contribution of CD4^+^ and CD8^+^ T cells to protection from FV challenge. CB6F1 mice were immunized twice with MCMV.env in a three-week interval, and depleted of CD4^+^ or CD8^+^ T cells five weeks after the first immunization. Mice were infected with FV one week later, and development of splenomegaly was monitored by twice-weekly palpation of the spleens (A). Viremia levels were determined in blood samples collected 10 days after FV infection (B); spleens were isolated three weeks after FV infection and spleen viral loads were analysed (C); pale symbols indicate mice that were sacrificed prior to the end of the experiment due to FV-induced disease in accordance with termination criteria, pale symbols do not represent actual values. The CD8^+^ T cell response to the Leader-Gag-derived GagL_85-93_ epitope was analysed ten days after FV challenge by MHC I tetramer staining, dots with black borders indicate mice that controlled FV-induced splenomegaly, dots with grey lines indicate mice that did not control FV-induced splenomegaly (D). The neutralizing antibody response in CD4^+^ or CD8^+^ cell depleted mice was analysed ten days after FV challenge (E). (F) Spearman correlation analysis was performed for neutralizing antibody levels in MCMV.env immunized, CD4^+^ cell-depleted mice before FV challenge infection with the viremia levels 10 days after FV infection. Each dot indicates an individual mouse, columns indicate mean (A, D) or median values (B, C, E), column whiskers indicate the standard error of the means (A), dotted lines indicate the limits of detection, grey lines (F) indicate the 95% confidence interval. Data were obtained in two (A–D) or three (E) independent experiments, using (A–C) 7 (unvaccinated), 8 (MCMV.env) or 10 mice (MCMV.env CD4 depleted, CD8 depleted) per group, (D) 5 (naïve), 6 (unvaccinated) or 8 mice (MCMV.env) per group or (E) 14 (unvaccinated, MCMV.env CD4 depleted) or 21 mice (MCMV.env) per group. Statistically significant differences compared to unvaccinated mice are indicated by *, significant differences compared to MCMV.env immunized, non-depleted mice are indicated by #, significant differences compared to naïve mice are indicated by † (*P* < 0.05, One Way ANOVA on Ranks, Dunn’s post test).

To analyse if CD8^+^ T cells are actually dispensable for control of the FV challenge after MCMV.env immunization, we performed depletion experiments where CD8^+^ T cells, or CD4^+^ T cells as control, were depleted from MCMV.env immunized mice starting one week before FV challenge infection. Surprisingly, depletion of either CD4^+^ or CD8^+^ cells resulted in loss of control over splenomegaly, with severely enlarged spleens from day 10 in contrast to non-enlarged or minimally enlarged spleens in MCMV.env immunized, non-depleted mice (Fig 9A). Similarly, when we analysed viral loads in plasma on day 10 after FV challenge and spleen viral loads on day 21 after FV challenge, we found that depletion of CD4^+^ cells as well as CD8^+^ cells resulted in high viral loads in most mice (Fig 9B and 9C).

The depletion of CD8^+^ T cells had a detrimental effect on MCMV.env-induced protection from FV challenge, even though the peptide stimulations gave no indication for the presence of Env-specific CD8^+^ T cells. This counterintuitive result could be explained by the fact that after FV challenge infection, also MCMV.env immunized mice mount a CD8^+^ T cell response against the GagL_85-93_ epitope, which is not part of the MCMV.env vaccine. When we analysed the GagL_85-93_-specific CD8^+^ T cell response ten days after FV infection, we found indeed that unvaccinated mice and MCMV.env immunized mice mounted a comparable GagL_85-93_-specific CD8^+^ T cell response (Fig 9D).

The development of disease in most CD4^+^ cell depleted mice underlines the importance of CD4^+^ T cells for the MCMV.env-mediated protection. To confirm the mechanistic role of MCMV.env-induced CD4^+^ T cells in providing help for the induction of potent antibody responses upon FV challenge infection, we compared the levels of neutralizing antibodies in MCMV.env immunized mice that were challenged with FV without further intervention, or after depletion of CD4^+^ cells, and found significantly reduced levels of neutralizing antibodies on day 10 after FV infection in mice depleted of CD4^+^ cells compared to MCMV.env immunized mice (Fig 9E). Only few of the CD4^+^ cell depleted MCMV.env immunized mice mounted a detectable neutralizing antibody response; importantly, those were the few CD4^+^ cell-depleted mice that were able to control the FV challenge (compare Fig 9B and 9C) and sera of these mice collected before FV challenge had also shown some neutralizing activity. While the overall correlation of neutralizing antibody titers before FV infection and viremia levels after FV infection was moderate (r = -0.3876; *P* = 0.2081; Fig 9F), the two mice that controlled the FV infection did indeed exhibit the highest neutralizing antibody levels before FV challenge, supporting the important role of antibodies in MCMV.env-mediated protection.

### Transfer experiments confirm the important role of antibodies in FV control after MCMV.env immunization

**Fig 10:**
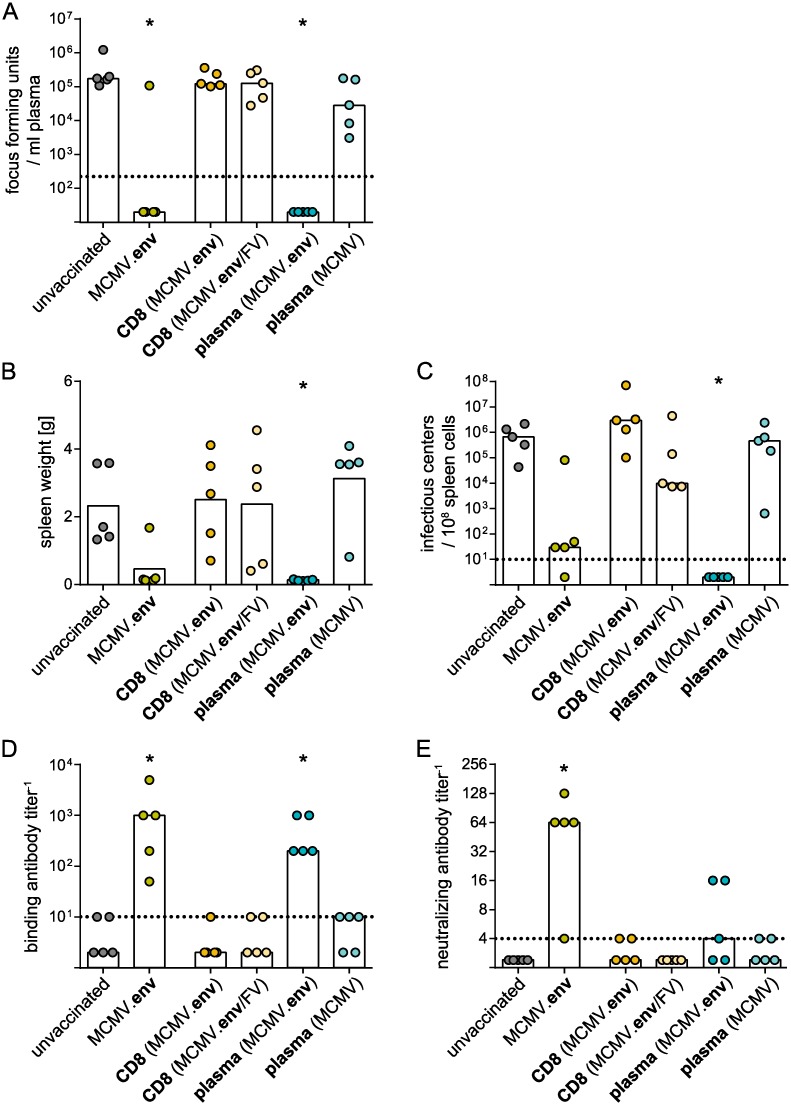
Transfer of plasma confers protection. Donor CB6F1 mice were immunized once with MCMV or MCMV.env and infected with 5 000 SFFU FV six weeks later. CD8^+^ T cells were isolated from spleens of MCMV.env immunized mice either six weeks after immunization, or two weeks after FV challenge infection and transferred into naïve recipient CB6F1 mice. Plasma was isolated from MCMV or MCMV.env immunized mice two weeks after FV challenge infection and transferred into naïve recipient CB6F1 mice. One day after the transfer of plasma or CD8^+^ T cells mice were infected with 5 000 SFFU FV. MCMV.env immunized mice and unvaccinated mice served as controls and were infected with 5 000 SFFU FV at the same time. Viral loads in plasma (A) was analysed ten days after FV challenge, spleen weights (B) and spleen viral loads (C) were analysed three weeks after FV challenge. Binding (D) and neutralizing antibody titers (E) were analysed ten days after FV challenge. Data were obtained in one experiment, each dot indicates one mouse, bars indicate median (A, C-E) or mean (B) values. Statistically significant differences compared to unvaccinated mice are indicated by * (*P* < 0.05, One Way ANOVA on Ranks, Dunn’s post test).

To corroborate our findings that showed the important role of antibodies in FV control after MCMV.env immunization and to confirm the absence of protective CD8^+^ T cells after MCMV.env immunization, we performed transfer experiments using CD8^+^ T cells isolated from MCMV.env immunized mice either before or after FV challenge infection, or using plasma isolated from control MCMV or MCMV.env immunized mice 14 days after FV challenge infection. As expected, transfer of CD8^+^ T cells did not result in significant control of FV infection, with viral loads in plasma (Fig 10A) and spleens (Fig 10C) that were not significantly reduced compared to unvaccinated mice. While transfer of plasma from control MCMV immunized mice collected after FV challenge had no significant impact on FV control, transfer of plasma from MCMV.env immunized mice on the other hand conferred complete protection to recipient mice, with undetectable viral loads in plasma and spleens (Fig 10A and 10C) and very low spleen weights (Fig 10B). An analysis of binding (Fig 10D) and neutralizing antibodies (Fig 10E) revealed similar levels of binding antibodies, and reduced levels of neutralizing antibodies, in recipients of MCMV.env immunized mouse-derived plasma compared to MCMV.env immunized mice. These results support the idea that MCMV.env immunized mice mount very potent antibody responses after FV challenge infection, which are able to mediate control over FV infection.

**Fig 11:**
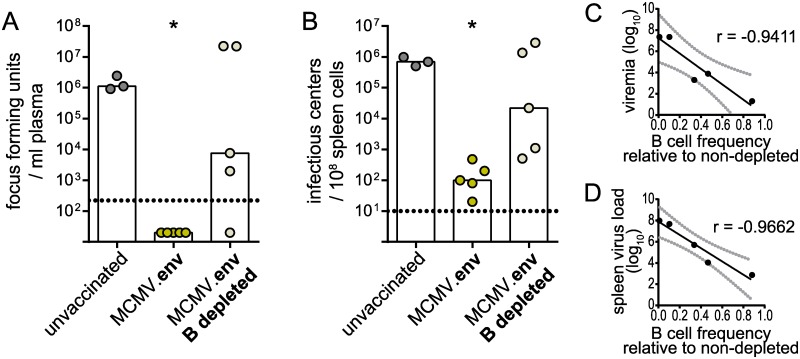
B cell depletion abrogates protection. CB6F1 mice were immunized once with MCMV.env and depleted of B cells by injection of an anti-CD20 antibody. Four days later, mice were infected with 5 000 SFFU FV. MCMV.env immunized mice without B cell depletion and unvaccinated mice served as controls. Viral loads in plasma were analysed ten days after FV infection (A), viral loads in spleens were analysed three weeks after FV challenge infection (B). B cell depletion efficacy was analysed ten days after FV infection in peripheral blood, and linear correlation analysis of B cell depletion efficacy and viremia (C) or spleen viral loads (D) was performed. Data were obtained in one experiment, each dot indicates one mouse, bars indicate median values, grey lines indicate 95% confidence intervals. Statistically significant differences compared to unvaccinated mice are indicated by * (*P* < 0.05, One Way ANOVA on Ranks, Dunn’s post test).

To furthermore prove the importance of the antibody response in MCMV.env immunization, we depleted immunized mice of B cells using a CD20-specific B cell depleting antibody. In contrast to MCMV.env immunized mice, mice that were immunized with MCMV.env and depleted of B cells before FV challenge infection mostly failed to control FV infection. Only one B cell depleted mouse was able to control viremia (Fig 11A), and all B cell depleted mice had higher viral loads in spleens than non-depleted mice going up to the same level as unvaccinated mice (Fig 11B). The B cell depletion did not work equally well in all mice; interestingly, when we performed correlation analyses, we found strong inverse correlations between the B cell frequency compared to non-depleted mice and the viral loads in plasma as well as spleens (viremia: r = -0.9411, *P* = 0.017; spleen virus load: r = -0.9662, *P* = 0.0074; Fig 11C and 11D).

Taken together, these results strongly support the important role of antibodies and B cells in MCMV.env mediated protection.

### MCMV.env immunization induces long-lasting protection from FV infection, but is not efficient in pre-existing MCMV immunity

The induction of long-lasting immunity is a pre-requisite for any vaccine. Therefore, we analyzed if the MCMV.env immunization would also protect from an FV challenge infection applied three months later.

**Fig 12:**
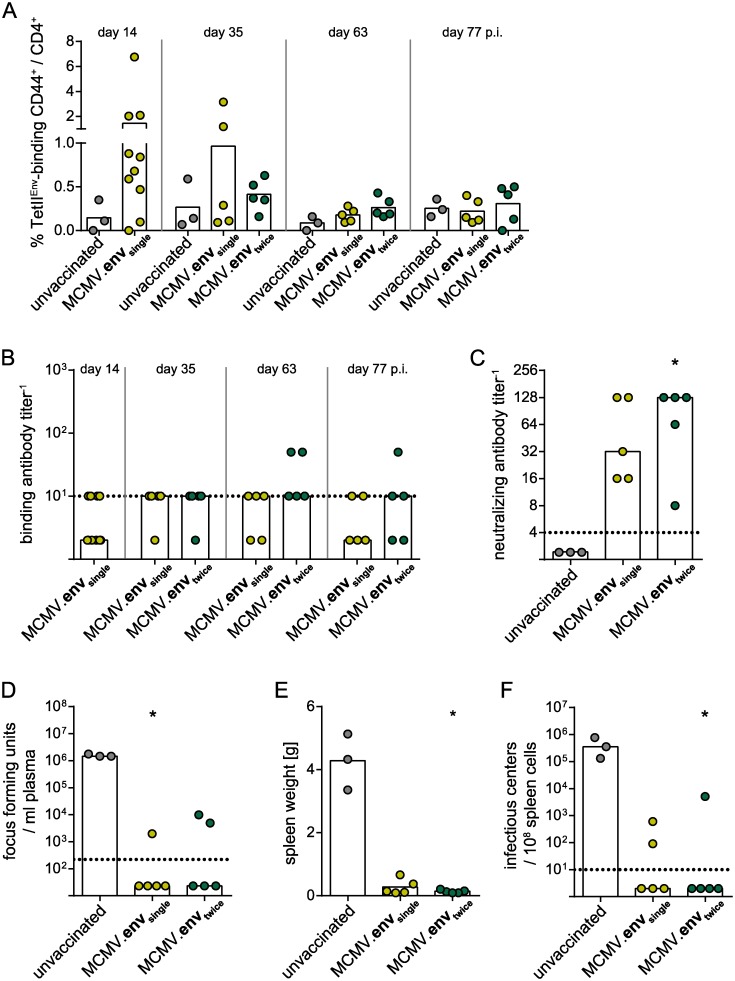
MCMV.env immunization confers long-lasting protection. CB6F1 mice were immunized once or twice in a three-week interval, and infected with 5 000 SFFU FV 12 weeks after the first immunization. The Env-specific CD4^+^ T cell response (A) and binding antibody titers (B) were monitored over time. The neutralizing antibody response (C) and viral loads in plasma (D) were analysed ten days after FV infection, spleen weights (E) and spleen viral loads (F) were analysed three weeks after FV infection. Data were obtained in one experiment, each dot indicates one mouse, bars indicate mean (A, E) or median values (B-D, F), dotted lines indicate the limits of detection. Statistically significant differences compared to unvaccinated mice are indicated by * (*P* < 0.05, One Way ANOVA on Ranks, Dunn’s post test).

CB6F1 mice were immunized once or twice as described before, and the development of FV-specific immune responses was monitored over time. The Env-specific CD4^+^ T cell response contracted quickly after the immunization to frequencies below 0.5%, and again we did not observe any impact of the second MCMV.env application (Fig 12A). The titers of F-MuLV-binding antibodies did not change significantly and were rather low during the whole observation period (Fig 12B). When mice were infected with FV three months after the initial immunization, they were able to mount strong neutralizing antibodies as observed in the previous experiments described above (Fig 12C), and controlled the FV infection tightly with low viral loads in plasma (Fig 12D), no splenomegaly (Fig 12E) and low spleen viral loads (Fig 12F).

**Fig 13:**
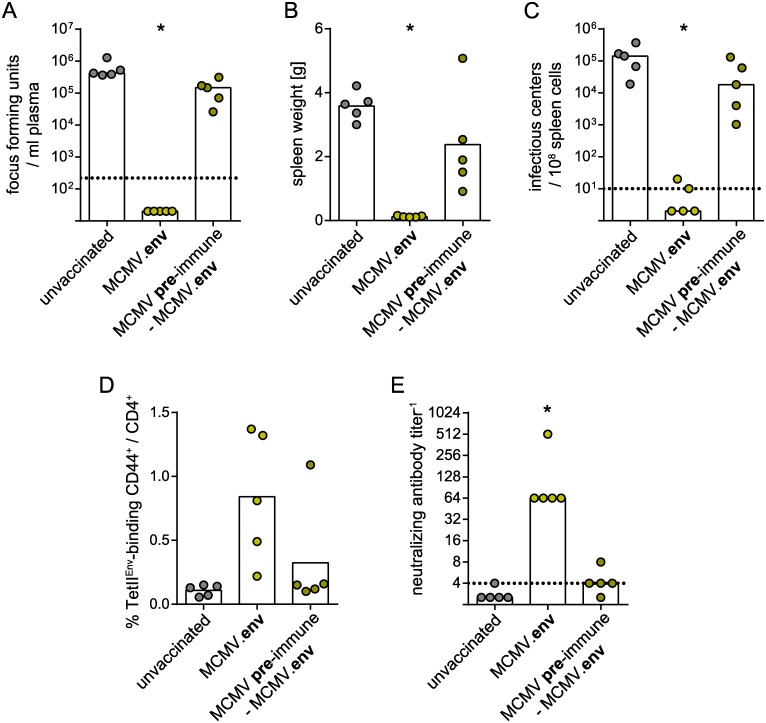
Immunization in pre-existing MCMV-specific immunity. CB6F1 mice were once immunized with MCMV.env, with or without one injection of non-transgene-encoding MCMV five weeks before. Six weeks after MCMV.env immunization, mice were infected with 5 000 SFFU FV. Viral loads in plasma were analysed ten days after FV infection (A), spleen weights (B) and spleen viral loads (C) were analysed three weeks after FV infection. The Env-specific CD4^+^ T cell response was analysed two weeks after MCMV.env immunization (D), F-MuLV neutralizing antibody titers were analysed ten days after FV infection (E). Data were obtained in one experiment, each dot indicates one mouse, bars indicate median (A, C, E) or mean values (B, D), dotted lines indicate the limits of detection. Statistically significant differences compared to unvaccinated mice are indicated by * (*P* < 0.05, One Way ANOVA on Ranks, Dunn’s post test).

These results show that protection conferred by MCMV.env immunization is long-lived. Another important consideration in the development of vector-based immunization strategies is the influence of pre-existing immunity against the vector. Therefore, we performed a vaccination experiment in mice that were infected with control MCMV five weeks before the MCMV.env immunization to induce MCMV immunity. When mice were infected with FV six weeks after the MCMV.env immunization, pre-immune mice were not able to control the FV infection and had high viral loads in plasma and spleens and developed splenomegaly (Fig 13A–13C). An analysis of the immune responses revealed a trend to reduced induction of Env-specific CD4^+^ T cells and a severely impaired induction of neutralizing antibodies upon FV challenge infection in MCMV pre-immune mice compared to pre-naïve MCMV.env immunized mice (Fig 13D and 13E).

Taken together, our results show that MCMV.env immunization leads to strong protection of highly susceptible mice from FV infection, which relies on a strong anamnestic neutralizing antibody response that fully expands only after FV infection and is not sufficient to prevent infection at challenge, therefore, vaccine-induced CD4^+^ T cells as well as intrinsic CD8^+^ T cells are equally required for control of FV challenge. While the strong protection mediated by MCMV.env was long-lasting, it was limited to MCMV pre-naive mice.

## Discussion

CMV-based vectors have been one of the most attractive vector systems of the last years, mainly because a RhCMV-based SIV vaccine was able to confer potent protection to rhesus macaques from SIV infection. We show here that in the FV mouse model, an MCMV-based vector encoding F-MuLV Env conferred strong protection to highly FV-susceptible mice. Protection correlated strongly with the ability of the mice to mount a rapid and strong anamnestic antibody response upon challenge, but not directly with the strength of the Env-specific CD4^+^ T cell response, even though the cytokine profile of the Env-specific CD4^+^ T cells suggest that they may have some direct antiviral activity and thus contribute to control of the FV infection.

It is a very interesting finding that MCMV.env immunization resulted in induction of strong CD4^+^ T cells as previous work with an MCMV vector encoding a tetanus toxin fragment had demonstrated potent antibody responses, but no appreciable cellular responses [8], similar to a RhCMV-based vaccine against Ebola virus that was tested in rhesus macaques [9]. The data presented here show in fact the strongest CD4^+^ T cell response to any FV vaccine that we have tested so far: adenovirus-based vectors as well as attenuated F-MuLV induced Env-specific CD4^+^ T cell responses that were far lower than the response shown here for MCMV.env immunization [40]. The antibody response to MCMV.env immunization, on the other hand, was rather low and strengthening the antibody response might be a promising strategy for further improvement of MCMV vector-based immunization efficacy. We did not detect any Env protein incorporated in MCMV.env virions, and the targeted incorporation of Env into the MCMV particle could possibly lead to improved Env-specific antibody responses in the same way as we could demonstrate for a so-called expression-display adenovirus-based vector that presents the F-MuLV Env gp70 protein on the capsid surface [32].

There is only a limited number of epitopes known in FV, and whereas the number of described FV-derived CD4^+^ T cell epitopes has increased in recent years [44], the Leader-Gag-derived GagL_85-93_ peptide is still the only confirmed CD8^+^ T cell epitope [43]. A CD8^+^ T cell epitope that was described for the Env protein [42] has never been confirmed in subsequent studies, and also our new data from MCMV.env immunized mice does not provide any indication for the presence of CD8^+^ T cells reactive to Env. Therefore, a strategy that might result in improved protection could be the incorporation of the Leader-Gag protein or the GagL_85-93_ epitope into an MCMV vector, as it has been demonstrated before that MCMV-based epitope vaccines were able to induce very potent immunogen-specific CD8^+^ T cell responses [6, 7]. However, we have shown that the GagL_85-93_ epitope is rather weak and can be sub-dominant to vector-derived epitopes [45], which might result in impaired GagL_85-93_-specific CD8^+^ T cell responses in the MCMV background. We have also shown before that Env suppresses CD8^+^ T cell responses to simultaneously or subsequently administered immunogens [40, 46]; interestingly, we did not observe a suppressive effect of Env on MCMV-specific CD8^+^ T cell responses, so it would be intriguing to see if CD8^+^ T cell responses that are suppressed in DNA or adenovirus-based immunization are also unaffected by Env in the MCMV background.

While the MCMV.env vaccine did not induce any Env-specific CD8^+^ T cells, we showed that the presence of CD8^+^ T cells at the time of challenge is required as mice mount an intrinsic, GagL_85-93_-specific CD8^+^ T cell response upon FV challenge that contributes to control. Interestingly, transfer of CD8^+^ T cells from MCMV.env immunized, FV-challenged mice did not confer protection to recipient mice, which may be attributable to the relatively low frequencies of GagL_85-93_-specific CD8^+^ T cells in the FV-challenged mice. In the past we have developed an adenovirus-based epitope vaccine which induced exclusively GagL_85-93_-specific CD8^+^ T cells and conferred strong protection from FV challenge [35], but there the frequency of GagL_85-93_-specific CD8^+^ T cells was ~ 10%, i.e. approximately five times as high as the frequency observed here in the MCMV.env immunized, FV-challenged mice. Furthermore, we infected mice with the high dose of 5 000 SFFU, which is not easily controlled by the highly FV-susceptible CB6F1 mice, demonstrating the stringency of our infection model and the potency of the MCMV.env vaccine.

One feature that makes CMV an interesting vaccine vector is its ability to establish a persistent infection, allowing for ongoing transgene expression and thereby acting as a self-boosting vaccine. To prevent strong activation of natural killer (NK) cells, we utilized an *m157* deleted MCMV for vector construction [41]; this modification did not result in an exacerbated pathology in the infected mice, but did allow for slightly elevated replication in the early phase of MCMV infection. This reduced activation of NK cells may also explain why, contrary to a previous report [47], we did not observe an effect of the control MCMV immunization on FV infection. Our challenge experiments showed that the outcome of an early FV challenge was far more variable than that of a late FV challenge, which we attribute to the higher neutralizing antibody levels that mice were able to mount after the late FV challenge. This delay in protection argues for the hypothesis that ongoing immunogen production by the MCMV.env vector promotes the development of protective immunity. On the other hand, antigen presenting cells of the B cell follicle are able to incorporate an antigen reservoir and to provide the antigen for B cell maturation for a prolonged time; therefore, experiments with a single-cycle MCMV would be necessary to confirm the supportive role of vaccine vector persistence. The development of a more attenuated or single-cycle MCMV might also be desirable for a translation into a CMV based vaccine for application in humans to increase the vaccine’s safety profile. Similarly, it would be interesting to analyse if different application routes result in comparable immune responses, as that would be necessary for a translation into vaccination of humans, and both questions shall be addressed in the future.

It has been demonstrated in the past using attenuated retrovirus-based vaccines that complex immune responses are necessary to confer full protection from retrovirus infection [20]; however, in immunization experiments with other vaccines, we could show that very potent vaccines can be generated that induce only partial immune responses. When we immunized mice with calcium phosphate nanoparticles encapsulating the immunodominant Env_123-141_ CD4^+^ T cell epitope and the GagL_85-93_ CD8^+^ T cell epitope, highly susceptible CB6F1 mice that we also used in the study presented now were mostly able to control FV-induced disease and exhibited significantly reduced spleen viral loads [29]. Mice that we immunized with an advanced scheme of adenovirus-based immunization vectors mounted robust CD4^+^ and CD8^+^ T cell responses but low neutralizing antibody responses upon immunization, but were strongly protected from FV-induced disease and had very low spleen viral loads around the detection limit [40]. Interestingly, mice that we immunized with Fv-1^b^-restricted, N-tropic F-MuLV (F-MuLV-N) that is highly attenuated in CB6F1 mice (Fv-1^b/b^) showed the strongest control over FV, but here the protection seemed to rely mostly on neutralizing antibodies, as no FV-specific CD8^+^ T cells and low CD4^+^ T cells were detectable after immunization [40]. As the F-MuLV-N immunization was superior even to the advanced adenovirus-based immunization, these findings highlight the importance of antibodies for protection from retrovirus infection, and the results obtained in the MCMV immunization study presented here are well in line with these results. In our model, the MCMV immunization did not seem to induce any FV-specific CD8^+^ T cell response, and protection was established with a delay after immunization, arguing for an important role of an antibody response that requires longer time to mature. Importantly, the MCMV-based vaccine allowed even the highly FV-susceptible mice used in this study to tightly control both FV-induced disease and FV loads; many mice displayed spleen viral loads below the detection limit of the immunocytochemical assay, and this is a level of protection that is comparable to the protection we observed after F-MuLV-N immunization under identical conditions [40], underlining the potency of our MCMV.env vaccine.

Our data show that immunization efficacy was severely affected by a prior infection with MCMV. Most reports on CMV-based immunization have not addressed the influence of pre-existing immunity; the studies using RhCMV vectors for immunization against SIV have been performed in naturally RhCMV infected macaques though, and found the vaccine to be highly effective [10]. In contrast to the vector design employed in the macaque studies, which induces strong and broad CD8^+^ T cell responses, our vector design that leads to strong CD4^+^ T cell and anamnestic antibody responses is restricted to MCMV-naïve vaccinees. It would have to be explored if a different design of our MCMV vector would allow for induction of transgene-specific immunity in pre-existing MCMV immunity. The expression of the immunogen under an immediate early promotor might result in earlier accumulation of immunogen than with our current MCMV.env construct and provide meaningful amounts of immunogen in spite of early clearance of MCMV.env infected cells. Also the incorporation of Env into the MCMV particle, as discussed above, might be useful to allow for the induction of Env-specific immunity in spite of pre-existing MCMV immunity. Strategies such as these should be developed and carefully evaluated to obtain MCMV vectors that are also highly efficacious in pre-existing MCMV immunity.

Overall, our results show that in our mouse model, MCMV-based vaccines prove highly effective tools for vaccination that lead to protection that is comparable in strength to the protection conferred by immunization with attenuated retrovirus, and highlight the importance of pursuing the development of CMV-based vectors further.

## Materials and methods

### Ethics statement

Mouse experiments were performed in accordance with the guidelines of the University Hospital Essen, Germany, the national animal protection law (Tierschutzgesetz (TierSchG)) and animal experiment regulations (Tierschutz-Versuchstierverordnung (TierSchVersV)), and the recommendations of the Federation of European Laboratory Animal Science Association (FELASA). The study was approved by the Northrhine-Westphalia State Office for Nature, Environment and Consumer Protection, Section 81 “Animal Research Affairs” (LANUV NRW, Düsseldorf, Northrhine-Westphalia, Germany; permit numbers 84–02.04.2014.A175 and 84–02.04.2017.A091).

### Cells and cell culture

A murine fibroblast cell line from *Mus dunni* [48] and the murine hybridoma cell line 720 [49] (both cell lines kindly provided by Dr. Kim J. Hasenkrug, NIAID, NIH, Hamilton, MT) were maintained in RPMI medium (Invitrogen/Gibco, Karlsruhe, Germany) supplemented with 10% heat-inactivated fetal bovine serum (Invitrogen/Gibco), 50 μg/ml gentamicin and 20 μg/ml ciprofloxacin. Primary mouse embryonic fibroblasts (MEF) were isolated according to described protocols [50]. Cells were maintained in a humidified 5% CO_2_ atmosphere at 37°C.

### Viruses

The recombinant mouse cytomegalovirus (MCMV; *Murid herpesvirus 1*) MCMV.env encoding Friend murine leukemia virus Env has been described before [51]. Briefly, MCMV.env was constructed by inserting an expression cassette containing the human CMV major immediate early promoter/enhancer and the F-MuLV Env coding sequence into the *m157* open reading frame of an MCMV with MCK2-repaired background; incorporation as well as retention of the transgene was confirmed by PCR. An *m157*-deleted MCMV vector without transgene was used as control. For *in vitro* and *in vivo* characterization, a wildtype MCMV [52] was used as control.

Uncloned, lactate dehydrogenase-elevating virus (LDV)-free FV stock was obtained from BALB/c mouse spleen cell homogenate (10%, wt/vol) 14 days post infection with a B-cell-tropic, polycythemia-inducing FV complex [53].

### Analysis of transgene expression and incorporation

For the analysis of transgene expression, MEF cells were infected with the MCMV vectors at an MOI of 5. Cells were collected 4, 24 and 48 hours after infection and cell lysates were subjected to SDS-PAGE and Western Blot analysis, probing with the F-MuLV gp70-specific hybridoma-derived antibody 720 [54], an MCMV pIE1-specific antibody (CROMA101; provided by Stipan Jonjić, University of Rijeka, Croatia) and an actin-specific antibody (Sigma-Aldrich, Munich, Germany). For the analysis of Env incorporation into vector particles, 2 × 10^5^ PFU MCMV.env or MCMV, or different amounts of F-MuLV particles as positive control (1 × 10^5^ FFU, 5 × 10^3^ FFU, or 1 × 10^3^ FFU) were subjected to SDS-PAGE and Western Blot analysis, using a polyclonal goat-anti-gp70 antibody (kindly provided by Dr. Christine Kozak, NIAID, NIH, Bethesda, MD). Detection of MCMV gB (15A12-H9; [50]) served as positive control.

### Mice

Female CB6F1 hybrid mice (BALB/c x C57BL/6 F1; H-2^b/d^ Fv1^b/b^ Fv2^r/s^ Rfv3^r/s^) and female BALB/c mice were purchased from Charles River Laboratories (Sulzfeld, Germany). All mice were used when they were between 8 and 9 weeks of age.

### Immunization

CB6F1 mice were immunized by intraperitoneal injection of 2 × 10^5^ plaque forming units of the recombinant MCMV vector. If the vaccine was applied repeatedly, the second immunization was applied three weeks later.

### FV challenge infection

CB6F1 mice were challenged by the intravenous injection of 5 000 spleen focus-forming units.

The development of FV-induced disease was monitored by palpation of the spleens of infected mice twice a week under general anaesthesia, and spleen sizes were rated on a scale ranging from 1 (normal spleen size) to 4 (severe splenomegaly) as described previously [55]. If mice showed overt signs of severe disease before the end of the experiment as rated by pre-determined termination criteria, they were euthanized and excluded from further analysis.

### Viremia assay

Ten days post challenge (p.c.), plasma samples from CB6F1 mice were obtained, and viremia was determined in a focal infectivity assay [56]. Serial dilutions of plasma were incubated with *M*. *dunni* cells for 3 days under standard tissue culture conditions. When cells reached ~100% confluence, they were fixed with ethanol, labeled with F-MuLV Env-specific MAb 720 [49], and then with a horseradish peroxidase (HRP)-conjugated rabbit anti-mouse Ig antibody (Dako, Hamburg, Germany). The assay was developed using aminoethylcarbazole (Sigma-Aldrich, Deisenhofen, Germany) as substrate to detect foci. Foci were counted, and focus-forming units (FFU)/ml plasma were calculated.

### Infectious center assay

21 days p.c., animals were sacrificed by cervical dislocation, the spleens were removed and weighed, and single-cell suspensions were prepared. Serial dilutions of isolated spleen cells were seeded onto *M*. *dunni* cells, and cells were incubated under standard tissue culture conditions for 3 days, fixed with ethanol, and stained as described for the viremia assay. Resulting foci were counted, and infectious centers (IC)/10^8^ spleen cells were calculated.

### Binding antibody ELISA

For the analysis of F-MuLV-binding antibodies, MaxiSorp ELISA plates (Nunc, Roskilde, Denmark) were coated with whole F-MuLV antigen (5μg/ml). After coating, plates were blocked with 10% fetal calf serum in PBS, and incubated with serum dilutions. Binding antibodies were detected using a polyclonal rabbit-anti-mouse HRP-coupled anti-Ig antibody and the substrate tetramethylbenzidine (TMB+; both Dako Deutschland GmbH, Hamburg, Germany). Sera were considered positive if the optical density at 450 nm was 3-fold higher than that obtained with sera from naïve mice.

### Complement-dependent F-MuLV-neutralizing antibody assay

To detect F-MuLV-neutralizing antibodies, serial dilutions of heat-inactivated plasma in PBS were mixed with purified F-MuLV and guinea pig complement (Sigma Aldrich, Munich, Germany), incubated at 37°C for 60 min, and then added to *M*. *dunni* cells that had been plated at a density of 7.5 x 10^3^ cells per well in 24-well plates the day before. Seventy-two hours later cells were stained as described for the viremia assay. Dilutions that resulted in a reduction of foci by 90% or more were considered neutralizing.

### Tetramer staining of F-MuLV-specific CD4^+^ T cells

F-MuLV-specific CD4^+^ T cells were analyzed in peripheral blood cells, lymph node or spleen cells two weeks after immunization or 21 days p.c.; erythrocytes were lysed before the staining when blood samples were used. Cells were stained with an allophycocyanin (APC)-coupled major histocompatibility complex (MHC) class II tetramer (containing the I-Ab-restricted F-MuLV Env_123-141_ epitope EPLTSLTPRCNTAWNRLKL [57]; kindly provided by the MHC Tetramer Core Facility of the National Institutes of Health, National Institute of Allergy and Infectious Diseases, Atlanta, GA), fluorescein isothiocyanate (FITC)–anti-CD11b, peridinin chlorophyll protein (PerCP)–anti-CD43, Brilliant Violet (BV) 510-anti-CD44, BV605-anti-CD4 (Becton Dickinson, Heidelberg, Germany) and Fixable Viability Dye eFluor 780 (eBioscience, Frankfurt, Germany). Data were acquired on an LSR II flow cytometer (Becton Dickinson, Mountanview, CA) and analyzed using FlowJo software (Tree Star, Ashton, OR).

### Tetramer staining of F-MuLV-specific CD8^+^ T cells

F-MuLV-specific CD8^+^ T cells were analyzed in PBMC ten days p.c.; cells were stained with a PE-coupled MHC I tetramer (containing the H2-D^b^-restricted F-MuLV Gag-Leader derived GagL_85-93_ epitope AbuAbuLAbuLTVFL in which the cysteine residues of the original peptide sequence have been replaced by amino-butyric acid (Abu) to prevent disulfide bonding [43]; MBL, Woburn, MA), PerCP-anti-CD43, BV450-anti-CD8, BV510-anti-CD44 (Becton Dickinson, Heidelberg, Germany) and Fixable Viability Dye eFluor 780 (eBioscience, Frankfurt, Germany). Data were acquired on an LSR II flow cytometer (Becton Dickinson, Mountanview, CA) and analyzed using FlowJo software (Tree Star, Ashton, OR).

### IFNγ ELISpot

For the analysis of vaccine-induced CD8^+^ T cells, spleens were isolated from mice 6 weeks after MCMV immunization or two weeks after FV infection, and depleted of CD4^+^ cells using the Miltenyi CD4^+^ T cell isolation kit (Miltenyi Biotec, Bergisch Gladbach, Germany). Cells were stimulated in an IFNγ ELISpot plate (384-well ImmunoSpot, C.T.L. Europe, Bonn, Germany) with pools of peptides derived from F-MuLV Env (6–8 peptides / pool; crude 18-mer peptides with 11 amino acid overlap covering the whole Env sequence were obtained from Peptides&Elephants, Henningsdorf, Germany) in the presence of 10 units/ml of IL2, using 10μg/ml of each peptide and 2.5 × 10^5^ cells per stimulation. Alternatively, cells were stimulated with a pool of MCMV derived peptides (M45_985-993_ (HGIRNASFI), M57_816-824_ (SCLEFWQRV), M102_446-455_ (SIVDLRFAVL), m139_419-426_ (TVYGFCLV) and m141_15-23_ (VIDAFSRL; [58])). Cells were stimulated for 48 hours and IFNγ foci were visualized according to the manufacturer’s instructions and counted using a BioReader-7000 Fz (Bio-Sys, Karben, Germany).

### Intracellular cytokine staining

For the analysis of cytokine production by MCMV-specific CD8^+^ T cells, peripheral blood cells were restimulated *in vitro* with the MCMV-derived peptides as indicated above for 6 hours in the presence of 2 μg/ml brefeldin A. Cells were stained with BV421-anti-CD8 (BioLegend), BV510-anti-CD44 (Becton-Dickinson), Fixable Viability Dye eFluor 780 (FVD-eF780; eBioscience, Frankfurt, Germany) and FITC-anti-interferon γ (IFNγ) (eBioscience).

For the analysis of cytokine production by Env-specific CD4^+^ T cells, peripheral blood cells were stimulated for 40 hours in the presence of 10 units/ml IL2 and a pool of the Env-derived peptides Env_123-141_ (EPLTSLTPRCNTAWNRLKL), Env_57-71_ (ETVWAISGNHPLWTW), Env_91-105_ (GLEYRAPYSSPPGPP), Env_415-430_ (KGSYYLVAPAGTMWAC), Env_267-281_ (PRVPIGPNPVLADQL) and Env_277-291_ (LADQLSFPLPNPLPK) at a concentration of 10 μg/ml of each peptide, followed by an additional incubation for 6 hours in the presence of 2 μg/ml brefeldin A. Cells were stained with BV605-anti-CD4 (BioLegend), BV510-anti-CD4, FVD-eF780, FITC-anti-IL10 (Invitrogen), PE/Dazzle594-anti-IFNγ (BioLegend), PE-Cy7-anti-IL17 (Invitrogen), APC-anti-IL4 (eBioscience), and eFluor450-anti-IL2 (eBioscience).

For the analysis of cytokine production by Env-specific CD8^+^ T cells or CD4^+^ T cells, spleen cells were stimulated *in vitro* with pools of peptides derived from F-MuLV Env (6–8 peptides / pool; crude 18-mer peptides with 11 amino acid overlap covering the whole Env sequence were obtained from Peptides&Elephants, Henningsdorf, Germany), combining three pools per stimulation (Env Pool^3^) at a concentration of 10 μg/ml per peptide in the presence of 10 units/ml Il2 for 40 hours, followed by an additional incubation for 6 hours in the presence of 2 μg/ml brefeldin A. Cells were stained with BV605-anti-CD4, BV650-anti-CD44 (BioLegend), AlexaFluor700-anti-CD8 (eBioscience), FVD-eF780, FITC-anti-IL10, PE/Dazzle594-anti-IFNγ, PE-Cy7-anti-TNFα (BioLegend), APC-anti-IL4, and eFluor450-anti-IL2 and BV510-anti-GzmB (Becton-Dickinson).

Data were acquired on an LSR II flow cytometer (Becton-Dickinson, Mountain View, CA) and analysed using FlowJo software (Tree Star, Ashton, OR).

### Transfer experiments

For the transfer of plasma, blood was collected from MCMV.env or MCMV control vector immunized mice 14 days after FV challenge infection, mixed with 10 U/ml heparin and cleared of cells by centrifugation. Plasma collected from one immunized mouse (~300 μl) was injected intravenously into one recipient mouse. For the transfer of CD8^+^ T cells, spleens were collected from MCMV.env immunized mice 6 weeks after immunization or 14 days after FV challenge infection, single cell suspensions were prepared and CD8^+^ T cells were isolated from 10^8^ total spleen cells by magnetic cell sorting (CD8^+^ T cell untouched isolation kit, Miltenyi, Bergisch-Gladbach, Germany). The CD8^+^ T cells isolated from one mouse were transferred into one recipient mouse by intravenous injection in 200 μl PBS with 50 U/ml heparin.

### *In vivo* depletion

For the depletion of CD4^+^ or CD8^+^ T cells, mice were injected on five consecutive days leading up to FV challenge infection with 250 μl of hybridoma-derived antibodies 191.1 or 169.4 [59], respectively, followed by injections every other day until day 14 after FV challenge. To control the depletion efficacy, small volumes of blood were collected, stained with antibodies PE-anti-CD4 and BV421-anti-CD8 after erythrocyte lysis, and analyzed by flow cytometry. The depletion efficacy was higher than 98%.

For the depletion of B cells, mice were injected once intravenously with 250 μg of the anti-CD20 antibody SA271G2 (BioLegend).

### Statistical analyses

Statistical analyses were performed using the software GraphPad Prism 6 (GraphPad Software, La Jolla, CA), testing with the Wilcoxon signed rank test for the comparison of two groups, the ordinary one-way analysis of variance (ANOVA) and Holm-Sidak post test or the Kruskal-Wallis one-way analysis of variance on ranks and Student-Newman-Keuls (equally sized groups) or Dunn’s (unequally sized groups) multiple comparison procedure for the comparison of three or more groups, or with Spearman ranked analysis for the determination of correlation.
